# Use of Digital Technology Among Older Adults in Poland With and Those Without Near Visual Impairment: Cross-Sectional Study

**DOI:** 10.2196/68947

**Published:** 2025-08-11

**Authors:** Adrian Lange, Natalia Lange, Kacper Jagiełło, Bogdan Wojtyniak, Tomasz Zdrojewski

**Affiliations:** 1 Department of Preventive Medicine and Education, Medical University of Gdańsk, M. Skłodowskiej-Curie 3a, 80-210, Gdansk, 80-210, Poland, 48 58 349 11 11; 2 Department of Population Health Monitoring and Analysis, National Institute of Public Health-National Institute of Hygiene, Warsaw, Poland

**Keywords:** digital inclusion, telemedicine, telehealth, visual impairment, older adults, technology access, Poland

## Abstract

**Background:**

The rapid evolution of digital technologies has transformed many aspects of daily life, offering substantial benefits for health and well-being through telemedicine and telehealth services. However, disparities in access to these technologies, particularly among older adults with visual impairments, remain a significant concern.

**Objective:**

This study aimed to examine the differences in access to and use of digital technologies between older adults in Poland with near visual impairment and those without. In addition, it explored how sociodemographic factors, such as education level and place of residence, interact with near visual impairment to influence digital technology access and usage.

**Methods:**

This cross-sectional analysis used data from the PolSenior2 project, a nationwide, multicenter survey conducted between 2018 and 2019. The sample included 5872 community-dwelling Polish adults aged 60 years and older, selected using a random, 3-stage, proportional sampling method, stratified by age and gender. Self-reported data on access to and usage of digital technologies, including smartphones, computers, and internet access, were collected. Near visual acuity was assessed using the Snellen chart for near vision.

**Results:**

Older adults with near visual impairment had significantly lower adjusted odds of owning and using digital devices compared to those without visual impairment. Specifically, the adjusted odds ratio of having and knowing how to use a smartphone was 0.62 (95% CI 0.46‐0.84), a computer 0.65 (95% CI 0.50‐0.86), and having internet access 0.64 (95% CI 0.48‐0.83), all indicating lower access among individuals with visual impairment. Furthermore, these individuals were less likely to use the internet for tasks such as searching for information about goods and services (adjusted odds ratio 0.65, 95% CI 0.49‐0.86).

**Conclusions:**

Older adults with near visual impairment in Poland face significant barriers in accessing and using digital technologies. These disparities highlight the need for targeted interventions to bridge the digital divide and improve digital inclusion for visually impaired seniors, ensuring they can benefit from the advantages of digital health solutions. Further research is required to develop and evaluate strategies to promote digital equity in this vulnerable population.

## Introduction

Globally, the swift progression of digital technology has significantly influenced various aspects of daily life, providing a multitude of benefits and conveniences. According to the World Health Organization (WHO), these advancements include improved health and well-being through the use of digital health services, often referred to as telemedicine or telehealth, which have become increasingly important [1]. Another study found that access to and use of digital technology can vary greatly among different population groups, particularly older adults and individuals with disabilities [2]. Furthermore, older adults with visual impairments encounter distinct challenges in accessing and using digital technologies, potentially exacerbating disparities in digital inclusion [2,3]. In addition, findings from a cross-sectional study indicate that older adults with visual impairments in the United States [3] and several European countries, such as Sweden [4] and Spain [5], are less likely than their peers without visual impairments to have access to digital technology. Numerous global studies indicate that this lack of access can result in increased isolation and diminished opportunities for these individuals to benefit from internet-based services and resources that could improve their quality of life [6-8]. Recent advancements in assistive technology, which use sensory modalities other than vision—such as screen readers, voice assistants, and text-to-speech—have the potential to mitigate some of these barriers [5,9-11].

In addition to challenges related to access to technology, the digital divide plays a crucial role in determining how effectively older adults engage with technology. The digital divide refers to the gap between individuals who can effectively use technology and those who cannot [12] and can be caused by a lack of accessible technology, limited digital literacy and competency, and inadequate internet accessibility [12]. Digital literacy refers to the basic skills required to use digital tools and access information [13], while digital competency involves advanced abilities to effectively and responsibly apply those tools in various contexts, such as problem-solving and collaboration [14]. Digital inclusion refers to the equitable access to, and effective use of, information and communication technologies that enhance individuals’ quality of life, bridging the digital divide by addressing factors such as infrastructure, affordability, and digital literacy [7]. According to the European Commission, the adoption and effectiveness of technological solutions vary across populations [15]. For instance, in Poland in 2023, challenges in adopting basic digital skills among the general population still persist [16]. Poland is a country where numerous older adults encounter challenges related to digital technology. Only 33.4% of people older than 65 years in Poland use a smartphone, according to the National Media Institute (2022), and 13.8% of older people use a laptop or notebook [17]. Individuals aged 65 years and older in Poland are generally poorly prepared to use the internet [18]. It is typically their children and grandchildren who assist them in using this medium, which would otherwise be beyond their reach [18]. A lack of basic user knowledge remains the main barrier for individuals older than 65 years who are willing to engage in meaningful interactions or make use of internet-based services [18].

Despite these challenges, there is no research specifically examining the use of digital technology by older adults with visual impairment in Poland. This gap in the literature underscores the need for further investigation. Therefore, the primary goal of our study was to examine how near visual impairment influences access to and use of digital technologies among older adults in Poland. In addition, we investigated how sociodemographic factors such as education level and place of residence interact with visual impairment to shape digital inclusion. By understanding these discrepancies, we can better address the unique needs of older adults with visual impairment in Poland and work towards greater digital inclusion for all.

## Methods

### Data Sources

The PolSenior2 project is a nationwide, multicenter, cross-sectional study carried out between 2018 and 2019. To ensure the representativeness of the older adult population in Poland, a random, 3-stage, proportional sampling method was used. This method, stratified by age and gender, facilitated the selection of 5987 Polish community-dwelling adults aged 60 years and older. Further details regarding the methodology are provided in another publication [19].

The study protocol encompassed the usage of 3 paper-based questionnaires, specialized geriatric tests, anthropometric and blood pressure measurements, and analyses of blood and urine samples. Trained nurses administered the medical and socioeconomic parts of the questionnaire via face-to-face interviews conducted during 3 home visits with the participants. Moreover, specific data points were obtained from the self-administered part of the questionnaire, completed autonomously by the respondents. All participants involved in the study provided written informed consent before their inclusion. The study protocol received approval from the Bioethics Committee of the Medical University of Gdansk (NKBBN/257/2017).

Initial ophthalmological screenings were conducted during the first visit as part of the medical section of the questionnaire, while data regarding digital technology usage were collected during the second visit as part of the socioeconomic section of the questionnaire. Data from 5872 participants were gathered and analyzed. A small fraction of respondents (1.9%) was excluded due to missing data. However, the absence of this information appeared to be random.

### Digital Technology Access

To assess whether individuals have and know how to use a cellphone, computer, internet, and smartphone, two questions were asked: (1) Is your household equipped with the above-mentioned devices or services? (2) Do you use these devices or services?

To determine the purpose of internet usage, we asked individuals to select one of the following options:

1. Sending and receiving mails
2. Searching for information (eg, about goods and services)
3. Use of internet banking
4. Use of instant messaging, for example, Skype, FaceTime, and Microsoft Messenger
5. Participating in chat rooms and using social networking sites, for example, Facebook
6. Searching for health care information
7. nternet shopping, ordering tickets, for example, cinema, theater, train, and airline tickets
8. Government websites for electronic handling of government inquiries (e-administration)

### Near Visual Impairment

Binocular near visual acuity (VA) with habitual correction was measured using the Snellen chart for near vision. This chart consisted of 8 sections of text with gradually increasing font size, marked from 1 (smallest print) to 8 (largest font). Functional near vision was measured at each participant’s preferred distance. For individuals who are unable to read or write, an “E” chart was used.

Based on the above data, respondents were categorized into the following groups:

1. Normal near vision: including individuals who could read lines 1‐4 from the standard distance (30‐40 cm).
2. Near visual impairment: including individuals who could not read lines 1‐4 from the standard distance (<30 cm or >40 cm), or read verses 5‐8 from any distance, or were unable to read any verses.

### Other Variables

Data on age, gender, living arrangement (alone/with others), place of residence (rural area or city with <20,000 population/larger city), education, household financial situation, and the number of comorbidities were also collected.

To determine the participants’ education level, the respondents were asked, “What is your current education level?” The possible answers were (1) no education, (2) incomplete primary education, (3) primary education, (4) middle school, (5) vocational high school, (6) high school, (7) 2-year college, (8) bachelor’s degree, “engineer’s degree,” and (9) master’s degree. To simplify the analysis, education levels (1)-(3) were classified as “primary or incomplete primary,” (4)-(6) were classified as “middle,” and (7)-(9) were classified as “higher.”

The financial situation of the household was assessed with the following question: “Which of the following sentences best describes the financial situation of your household?” The possible answers were (1) “We live comfortably without having to save for special purchases,” (2) “We live frugally and we have enough to cover all of our expenses,” (3) “We need to put money aside to save for special purchases,” (4) “We have enough money for basic needs such as food and clothing,” (5) “We only have enough money for food,” and (6) “We don’t have enough money even to meet our basic needs.” For consistency of the results, the responses were grouped into respective categories: Can easily afford everything (1), Can afford everything but only when saving (2), and Self-reported poverty (3)-(6).

We also considered comorbid conditions, including heart attack, heart failure, arterial hypertension, diabetes, stroke, dementia, chronic obstructive airway disease, and cancer, to get a better assessment of each group.

Diabetes mellitus was noted when a patient declared that it was previously diagnosed, if fasting glucose was ≥126 mg/dL, or if the use of hypoglycemic drugs was reported.

Hypertension was diagnosed if the average blood pressure values from 2 measurements during each visit were equal to or greater than 140 mm Hg (systolic blood pressure) and/or 90 mm Hg (diastolic blood pressure) or if the patient was taking hypotensive drugs over the past 2 weeks because of an earlier diagnosis of hypertension.

We assessed dementia using the Mini–Mental State Examination (MMSE) with the Mungas correction. Participants scoring 23 points or less on the MMSE were classified as having dementia.

Comorbidities, including heart attack, heart failure, chronic obstructive pulmonary disease, and cancer, were identified based on self-reported data. In addition, heart failure was verified through medical documentation.

### Statistical Analysis

Statistical analyses were performed using SAS 9.4 TS Level 1M5 (SAS Institute) and the R version 3.6.3 (R Foundation for Statistical Computing). The results were presented as percentages or percentages with 95% CIs. To compare the proportions, the chi-square test was applied. Sampling weights were included in statistical calculations to account for the complex survey design using the R survey package. The poststratification procedure was used to match the age–gender sample distribution to the population of Poland. Multivariable logistic regression models were created to identify associations of near visual impairment with technological environment outcomes. Every model was adjusted for age, gender, living arrangements, education, comorbidities, and income. In addition, to check for effect modification of near visual impairment by the socioeconomic characteristics of the respondents, our models included interaction terms of factors such as gender, level of education, living arrangements, place of residence, and visual impairment. For all statistical analyses, the level of significance was set at .05.

### Ethical Considerations

This study was conducted in accordance with the ethical standards of the Declaration of Helsinki and was approved by the Bioethics Committee of the Medical University of Gdańsk (approval number: NKBBN/183/2017, issued on October 17, 2017). All participants provided written informed consent prior to participation. Participation was voluntary, and respondents were informed of their right to withdraw from the study at any time without any consequences. Personal data were anonymized, and all information collected was handled with strict confidentiality in accordance with applicable data protection laws. No financial or material compensation was provided to participants for their involvement in the study.

## Results

A total of 2738 out of 5872 (46.63%) participants exhibited near visual impairment. (Table 1). The mean age of individuals with near visual impairment was 76.8 years, which is higher than the mean age of 73.1 years for those without such impairment (Table 1). Furthermore, adults with near visual impairment were less likely to have attained middle or higher education levels compared to their counterparts without near visual impairment. In addition, respondents with near visual impairment reported higher rates of comorbidities and more frequently experienced poverty compared with those without the impairment (Table 1).

**Table 1.** Demographics and characteristics by near visual impairment status.

Demographic characteristics	Near visual impairment (N=2738)	Normal vision (N=3134)	*P* value
Age, mean (SD)	76.8 (9.7)	73.1 (8.7)	<.001
Gender			.60
Overall, N	2738	3134	
Women, n (%)	1380 (50.4)	1602 (51.1)	
Men, n (%)	1358 (49.6)	1532 (48.9)	
Living arrangements			.82
Overall, n	2598	3007	
With others, n (%)	2042 (78.6)	2371 (78.8)	
Alone, n (%)	556 (21.4)	636 (21.2)	
Education			<.001
Overall, n	2684	3071	
Higher, n (%)	384 (14.3)	617 (20.1)	
Middle, n (%)	1327 (49.4)	1778 (57.9)	
Primary, n (%)	973 (36.3)	676 (22.0)	
Comorbidities			<.001
Overall, n	2588	3067	
0‐1, n (%)	1105 (42.7)	1597 (52.1)	
2, n (%)	794 (30.7)	864 (28.2)	
3, n (%)	417 (16.1)	397 (12.9)	
≥4, n (%)	272 (10.5)	209 (6.8)	
Income			.02
Overall, n	2624	3045	
Can easily afford everything, n (%)	478 (18.2)	595 (19.5)	
Can afford everything but only when saving, n (%)	1393 (53.1)	1674 (55.0)	
Self-reported poverty, n (%)	753 (28.7)	776 (25.5)	

Other variables, including gender and living arrangement, were comparable between the 2 groups (those with near visual impairment and those without near visual impairment) (Table 1).

The prevalence of having and knowing how to use cell phones, smartphones, computers, and access to the internet was lower among older adults with near visual impairment compared to their peers with normal vision (Table 2). Furthermore, in terms of the various purposes of internet usage, individuals with near visual impairment exhibited a lower engagement in every type of digital activity (Table 2).

**Table 2.** Weighted prevalence of technological environment outcomes by near visual impairment status.

Technological environment outcomes	Near visual impairment, OR^a^ (95% CI)	Normal vision, OR (95% CI)
Has and knows how to use the following		
Cellphone	80.9 (78.3‐83.5)	89.2 (86.6‐91.7)
Smartphone	15.7 (12.3‐19)	27.5 (23.9‐31)
Computer	36.9 (33.2‐40.5)	51.5 (47.4‐55.5)
Internet access	38.0 (34.2‐41.8)	53.6 (49.2‐58.0)
Internet activities		
Sending and receiving mails	15.2 (12.4‐18.1)	26.0 (21.9‐30.1)
Searching for information (eg, about goods and services)	25.6 (22.1‐29.1)	40.8 (36.0‐45.6)
Use of internet banking	13.0 (10.6‐15.4)	23.6 (20.0‐27.3)
Use of instant messaging for example, Skype, FaceTime, and Microsoft Messenger	11.0 (8.8‐13.2)	16.5 (13.7‐19.3)
Participating in chat rooms and using social networking sites for example, Facebook	5.9 (4.5‐7.3)	10.6 (8.1‐13.0)
Searching for health care information	15.9 (13.4‐18.5)	25.4 (22.3‐28.6)
Internet shopping, ordering tickets, for example, cinema, theater, train, and airline tickets	6.2 (4.7‐7.8)	13.5 (11.1‐15.9)
Government websites for electronic handling of government inquiries (e-administration)	4.0 (2.7‐5.3)	8.3 (5.8‐10.7)

a OR: odds ratio.

In a multivariable logistic regression analysis (Table 3), near visual impairment was associated with lower odds of having and knowing how to use a smartphone (odds ratio [OR] 0.62, 95% CI 0.46‐0.84), a computer (OR 0.65, 95% CI 0.50‐0.86), and access to the internet (OR 0.64, 95% CI 0.48‐0.83) compared with no visual impairment. Near visual impairment was also associated with lower odds of using the internet for searching for information (eg, about goods and services; OR 0.65, 95% CI 0.49‐0.86) compared with no visual impairment. No other associations were noted between near visual impairment status and digital experiences, such as sending and receiving emails, internet banking, searching for health care information, using instant messaging (eg, Skype, Facetime, and Microsoft Messenger), participating in chat rooms, using social networking sites (eg, Facebook), internet shopping or ordering tickets (eg, cinema, theater, train, airline, and tickets) or using government websites for electronic handling of government inquiries (e-administration) (Table 3).

**Table 3.** Multivariable logistic regression: associations of near visual impairment with technological environment outcomes.

Technological environment outcomes	Adjusted OR^a^ (95% CI)	*P* value
Has and knows how to use the following		
Cellphone	0.81 (0.56-1.17)	.26
Smartphone	0.62 (0.46-0.84)	.002
Computer	0.65 (0.50-0.86)	.002
Internet access	0.64 (0.48-0.83)	.001
Internet activities		
Sending and receiving mails	0.73 (0.53-1.0)	.005
Searching for information (eg, about goods and services)	0.65 (0.49-0.86)	.002
Use of internet banking	0.81 (0.58-1.13)	.22
Use of instant messaging, for example, Skype, Facetime, and Microsoft Messenger	1,04 (0.74-1.47)	.81
Participating in chat rooms and using social networking sites, for example, Facebook	0.96 (0.62-1.48)	.85
Searching for health care information	0.81 (0.60 to 1.09)	.17
Internet shopping, ordering tickets, for example, cinema, theater, train, and airline tickets	0.96 (0.65-1.43)	.86
Government websites for electronic handling of government inquiries (e-administration)	0.84 (0.52-1.35)	.46

a OR: odds ratio

The impact of near visual impairment on internet access among older adults in Poland was significant. The findings indicated that the odds of using the internet were substantially lower for individuals residing in rural areas or cities with populations under 20,000. The negative impact of visual impairment was modified by place of residence and was smaller in rural areas and small towns than in larger cities. This was demonstrated by the interaction term which yielded an OR of 1.41 (95% CI 1.06‐1.87; *P*=.02; Multimedia Appendix 1).

A similar effect modification of near visual impairment by place of residence was found for searching for information. Specifically, near visual impairment reduced the odds of searching for information less among individuals living in villages or cities with populations less than 20,000, compared with those in larger cities, and this result was statistically significant (OR 1.44, 95% CI 1.06‐1.97; *P*=.02). Conversely, near visual impairment decreased the odds of internet shopping more among individuals living in rural areas or cities with populations less than 20,000 compared to those in larger cities (interaction term: near visual impairment × place of residence – village or city with <20,000 population; OR 0.60, 95% CI 0.37‐0.99; *P*=.045). (Multimedia Appendix 1)

Furthermore, the effect modification of near visual impairment by education level was statistically significant for searching for health information, internet banking, and social networking at borderline significance (*P*=.06). Individuals with visual impairment and a lower level of education were less likely to be involved in these activities than better educated individuals with the same health problem (Multimedia Appendix 1). The analysis indicated that there were no statistically significant interactions between visual impairment and socioeconomic factors, such as gender and living arrangements, in relation to any aspect of digital technology access considered in our study (Multimedia Appendix 1).

## Discussion

### Principal Findings

The findings from our study revealed significant associations between near visual impairment and decreased digital engagement among older adults in Poland. Older adults with near visual impairment were less likely to own and know how to use smartphones, computers, and have access to the internet. This trend extended to digital experiences, where those with near visual impairment engaged less frequently in internet activities such as searching for information (eg, about goods and services).

However, the multivariable logistic regression analysis showed a lack of association between near visual impairment and the use of instant messaging, sending and receiving emails, internet banking, searching for health care information, participation in chat rooms, social networking, internet shopping or ordering tickets, or accessing government websites for e-administration. This may suggest that certain digital activities might be less influenced by visual impairment, possibly due to the availability of assistive technologies or the lower complexity of these tasks.

Moreover, our findings were consistent with international research, demonstrating that older adults, especially those with disabilities, encounter substantial obstacles in accessing digital technology [2-4,20]. A study conducted in the United States reported even greater disparities, with near visual impairment being associated with lower odds of having and knowing how to use a cell phone (OR 0.56, 95% CI 0.36‐0.87), a computer (OR 0.57, 95% CI 0.44‐0.75), or a tablet (OR 0.65, 95% CI 0.52‐0.81), sending messages by email or text (OR 0.56, 95% CI 0.40‐0.77), and accessing the internet beyond email or text messaging (OR 0.59, 95% CI 0.44‐0.80) compared with those without near visual impairment [3]. Near visual impairment; however, was not associated with any digital health– or non–health-related experiences such as visiting social network sites, visiting with family or friends on video calls [3]. Also, another study from the United States showed that impairments in vision were associated with decreased usage of email, text messaging, and the internet [2].

Research conducted in Sweden revealed that participants with visual impairments reported lower usage of digital identification and a tendency to avoid booking health care appointments via internet-based platforms compared to participants without visual impairments and those with other types of impairments, such as neurological, musculoskeletal, or hearing impairments [4].

According to many studies, visual impairments pose substantial barriers to digital inclusion, limiting the ability of affected individuals to benefit from internet-based resources and services [2-4,20]. Barriers include policies that do not explicitly support accessible design, webpage designs that are incompatible with assistive devices, and insufficient financial resources to acquire appropriate assistive technology [21]. However, contrary to common misconceptions, modern smartphones are highly accessible to individuals with visual impairment. These devices offer advanced features such as sound, haptics, gestures, high contrast ratios for colors, and the ability to increase text size by at least 200% [5,10,11,22,23]. Despite these accessible features, the adoption of smartphones and other assistive technologies remains limited among older adults with near visual impairment [24,25]. This is largely due to several barriers, including cost [25], limited awareness [18], and usability issues [26].

The cost of assistive technologies remains a significant obstacle for many individuals, particularly those with low socioeconomic status [27]. Even when these technologies are available, many individuals with visual impairment are unaware of their existence or how they can enhance digital engagement. Another study found that only 20% of older adults with visual impairment were aware of the availability of assistive technologies, and even fewer—about 8%—had a good understanding of how to use them effectively [18].

Therefore, it is crucial to address these barriers by enhancing awareness and providing training on how to use assistive technologies. Such efforts should involve health care professionals, educators, and the general public, ensuring that individuals with visual impairments are better equipped to take advantage of the accessibility features built into modern smartphones and other digital devices.

Furthermore, these efforts align with the WHO’s Global Strategy on Digital Health, which emphasizes the need for inclusive digital health systems that cater to all populations, including individuals with disabilities [1]. The WHO strategy highlights the importance of accessible design, digital literacy training, and policy interventions to bridge health-related digital divides [1]. Our study underscores the necessity of integrating these principles into national digital health initiatives to ensure that older adults in Poland with near visual impairment are not excluded from the benefits of digital health care.

To foster digital inclusion among older adults with near visual impairment, policy makers in Poland should implement comprehensive strategies that include digital literacy programs, subsidized assistive technologies, and design considerations for inclusive technology. Digital literacy programs should be tailored to older adults with visual impairments, focusing on improving digital skills, awareness of assistive technologies, and practical applications of digital tools in daily life. Subsidized assistive technologies are essential to overcoming financial barriers, ensuring access to screen readers, magnification software, and smart devices with accessibility features [10]. Public awareness campaigns are also crucial in raising awareness about available assistive technologies and digital accessibility features, helping older adults with near visual impairment recognize the benefits of engaging with digital platforms [28]. In addition, our results highlighted significant interaction effects between near visual impairment and both place of residence and education level on various internet-based activities, indicating that the barriers posed by visual impairment were compounded by certain sociodemographic factors. Rather unexpectedly, it significantly reduced the odds of accessing the internet and searching for information less in smaller communities (<20,000 population) compared with larger ones. Conversely, its impact on internet shopping was more evident in smaller communities. Individuals with visual impairment often face significant challenges in accessing and navigating physical stores [29], especially those residing in rural areas and small towns. This demographic is particularly disadvantaged due to the limited availability of retail establishments in those regions, compounded by the difficulties they encounter within store environments. Consequently, there is a heightened demand for internet shopping among this group. The convenience and accessibility of e-commerce platforms provide a vital alternative, enabling visually impaired persons to procure necessary goods and services without the obstacles associated with traditional brick-and-mortar stores. This reliance on internet shopping underscores the need for inclusive digital solutions that cater to their specific needs and preferences. This seems to be a global situation. For example, one study [30] showed that internet shopping is an integral component of the daily lives of people with disabilities in China, particularly for the visually impaired. However, another study showed that rural areas often lack the necessary infrastructure and reliable internet connectivity, posing significant challenges for residents in accessing digital resources and services [31]. This digital divide hinders the ability of rural inhabitants to benefit from internet platforms, further exacerbating the difficulties faced by individuals with visual impairment in these regions. However, according to the GUS, a Polish Census Bureau, the penetration of internet access is quite high in all of Poland, reaching 93,3% of households in 2022, with an almost even spread among cities and rural areas, big cities: 94.4%, small cities: 92,3% and rural areas: 93,2% [32,33].

In addition, individuals with visual impairments and lower levels of education are less likely to engage in certain internet-based activities, such as searching for health information, internet banking, and social networking, even when they experience the same health issues as those with higher education. Another study also highlights the disparities in the benefits of educational attainment for the visually impaired population compared to those without disabilities [34]. People with lower education levels often struggle to develop digital literacy and technical skills, which can limit their ability to participate in internet-based activities [35].

Our study contributes to the understanding of the digital divide by examining how visual impairment, combined with sociodemographic factors, exacerbates disparities in digital engagement. While the digital divide is often framed as a matter of access to digital technologies, our findings highlight that it also involves challenges related to effective usage. Older adults with near visual impairment face significant barriers in accessing and using digital platforms, particularly when factors such as place of residence and education level intersect with their disability. Our findings emphasize the need for targeted policy measures that align with broader digital inclusion frameworks, ensuring that technological advancements are accessible to all, regardless of disability or sociodemographic background.

### Strengths and Limitations

The strengths of this study include a large, representative sample and a comprehensive assessment of access and usage of digital technology. However, several limitations should be noted. First, the cross-sectional design precludes causal inferences regarding the relationship between visual impairment and digital technology usage. Second, data collection was conducted before the COVID-19 pandemic. In addition, our study did not assess distance visual impairment. This limitation arises from the cohort study design, where a comprehensive assessment of distance vision was difficult to implement. While half of the participants were tested using the Snellen chart for distance vision with an optotype of 0.1, the focus was placed on near vision as it is more directly relevant to the use of digital devices, such as smartphones and computers, which were central to our study. Moreover, our study focused exclusively on community-dwelling older adults, excluding institutionalized individuals. Finally, as digital technology use was self-reported, there is a potential for recall bias and social desirability bias, which may have influenced participants’ responses. Despite the precautions taken to minimize inaccuracies in the data, including face-to-face interviews by trained nurses and the involvement of caregivers where necessary, the reliance on self-reported data still presents limitations. Participants may have misreported or misunderstood certain questions, which could lead to residual inaccuracies in the dataset.

### Conclusions

In Poland, older adults with near visual impairment encounter substantial challenges in accessing and using digital technologies. Addressing these disparities is essential to foster greater digital inclusion to improve the quality of life for older adults with visual impairment in the country.

There is a need for targeted interventions to enhance digital inclusion for older adults with near visual impairment in Poland. Efforts should focus on improving digital literacy, enhancing the accessibility of digital devices and services, and providing tailored support to individuals with visual impairment. Such initiatives are essential to ensure that all older adults can fully participate in the increasingly digital society, thereby enhancing their quality of life and reducing social isolation. Further research is needed to explore the specific barriers faced by older adults with visual impairment and to develop effective strategies to bridge the digital divide.

## Supplementary material

10.2196/68947 Multimedia Appendix 1 Multivariable logistic regression model for selected technological environment outcomes among older adults in Poland, including interactions with selected socioeconomic factors (sex, educational level, place of residence, and living arrangements).

## Abbreviations

MMSE: Mini–Mental State Examination
OR: odds ratio
VA: visual acuity
WHO: World Health Organization
