# Supplementary material for: Use of Digital Technology Among Older Adults in Poland With and Those Without Near Visual Impairment: Cross-Sectional Study
Source: J Med Internet Res. 2025 Aug 11;27:e68947. doi: 10.2196/68947 (PMC12338959; doi:10.2196/68947)
Supplement: Multimedia Appendix 1 [file jmir-v27-e68947-s001.docx]

### Table S1. Multivariable Logistic Regression Model for Selected Technological Environment Outcomes among Older Adults in Poland, Including Interactions with Selected Socioeconomic Factors ( Sex, Educational Level, Place of Residence, Living Arrangements)

***Has and knows how to use cellphone***

| Variables | Estimate | OR | lower.CI | upper.CI | Z-value | P-value |
| --- | --- | --- | --- | --- | --- | --- |
| Near visual impairment | -0.21 | 0.81 | 0.56 | 1.17 | -1.12 | .263 |
| Women | -0.39 | 0.68 | 0.53 | 0.86 | -3.23 | .001 |
| Age | -0.09 | 0.91 | 0.91 | 0.92 | -18.95 | <.001 |
| Educational level - primary | -0.58 | 0.56 | 0.44 | 0.72 | -4.58 | <.001 |
| Income - afford when saving | -0.10 | 0.90 | 0.72 | 1.14 | -0.81 | .416 |
| Income - difficulties paying | -0.26 | 0.77 | 0.60 | 0.99 | -2.06 | .039 |
| Comorbidities | -0.06 | 0.94 | 0.88 | 1.01 | -1.74 | .083 |
| Living alone | 0.12 | 1.13 | 0.85 | 1.50 | 0.80 | .426 |
| Village or Small Town (<20,000 Residents) | -0.62 | 0.54 | 0.42 | 0.69 | -4.92 | <.001 |
| Near Visual Impairment x Sex (Women) | 0.12 | 1.13 | 0.82 | 1.56 | 0.71 | .476 |
| Near Visual Impairment x Educational Level (Primary) | -0.03 | 0.97 | 0.68 | 1.38 | -0.16 | .877 |
| Near Visual Impairment x Place of Residence (Village or Small Town (<20,000 Residents) | 0.04 | 1.04 | 0.74 | 1.46 | 0.21 | .834 |
| Near Visual Impairment x Living Arrangements (Alone) | -0.22 | 0.80 | 0.54 | 1.18 | -1.10 | .271 |

***Has and knows how to use smartphone***

| Variables | Estimate | OR | lower.CI | upper.CI | Z-value | P-value |
| --- | --- | --- | --- | --- | --- | --- |
| Near visual impairment | -0.47 | 0.63 | 0.46 | 0.84 | -3.07 | .002 |
| Women | -0.39 | 0.68 | 0.55 | 0.83 | -3.70 | <.001 |
| Age | -0.10 | 0.90 | 0.89 | 0.92 | -16.65 | <.001 |
| Educational level - primary | -1.15 | 0.32 | 0.25 | 0.39 | -10.36 | <.001 |
| Income - afford when saving | -0.63 | 0.53 | 0.44 | 0.64 | -6.43 | <.001 |
| Income - difficulties paying | -0.85 | 0.43 | 0.34 | 0.54 | -6.91 | <.001 |
| Comorbidities | -0.05 | 0.95 | 0.88 | 1.02 | -1.41 | .159 |
| Living alone | -0.13 | 0.88 | 0.68 | 1.14 | -0.95 | .342 |
| Village or Small Town (<20,000 Residents) | -0.62 | 0.54 | 0.44 | 0.66 | -5.86 | <.001 |
| Near Visual Impairment x Sex (Women) | 0.00 | 1.00 | 0.71 | 1.41 | 0.01 | .991 |
| Near Visual Impairment x Educational Level (Primary) | 0.29 | 1.34 | 0.93 | 1.91 | 1.58 | .114 |
| Near Visual Impairment x Place of Residence (Village or Small Town (<20,000 Residents) | -0.06 | 0.94 | 0.66 | 1.34 | -0.35 | .723 |
| Near Visual Impairment x Living Arrangements (Alone) | -0.09 | 0.91 | 0.58 | 1.45 | -0.37 | .712 |

***Has and knows how to use computer***

| Variables | Estimate | OR | lower.CI | upper.CI | Z-value | P-value |
| --- | --- | --- | --- | --- | --- | --- |
| Near visual impairment | -0.43 | 0.65 | 0.50 | 0.85 | -3.10 | .002 |
| Women | -0.37 | 0.69 | 0.57 | 0.83 | -3.96 | <.001 |
| Age | -0.11 | 0.90 | 0.89 | 0.90 | -23.87 | <.001 |
| Educational level - primary | -1.51 | 0.22 | 0.18 | 0.27 | -16.11 | <.001 |
| Income - afford when saving | -0.56 | 0.57 | 0.48 | 0.68 | -6.10 | <.001 |
| Income - difficulties paying | -0.92 | 0.40 | 0.32 | 0.49 | -8.53 | <.001 |
| Comorbidities | -0.06 | 0.94 | 0.89 | 1.00 | -2.06 | .039 |
| Living alone | -0.47 | 0.63 | 0.50 | 0.79 | -4.04 | <.001 |
| Village or Small Town (<20,000 Residents) | -0.73 | 0.48 | 0.40 | 0.58 | -7.88 | <.001 |
| Near Visual Impairment x Sex (Women) | 0.10 | 1.11 | 0.83 | 1.47 | 0.68 | .496 |
| Near Visual Impairment x Educational Level (Primary) | 0.04 | 1.04 | 0.78 | 1.38 | 0.29 | .774 |
| Near Visual Impairment x Place of Residence (Village or Small Town (<20,000 Residents) | 0.27 | 1.31 | 0.98 | 1.74 | 1.84 | .066 |
| Near Visual Impairment x Living Arrangements (Alone) | -0.08 | 0.92 | 0.64 | 1.34 | -0.43 | .666 |

***Has and knows how to use internet***

| Variables | Estimate | OR | lower.CI | upper.CI | Z-value | P-value |
| --- | --- | --- | --- | --- | --- | --- |
| Near visual impairment | -0.45 | 0.64 | 0.49 | 0.84 | -3.28 | .001 |
| Women | -0.33 | 0.72 | 0.60 | 0.86 | -3.48 | .001 |
| Age | -0.12 | 0.89 | 0.88 | 0.90 | -24.87 | <.001 |
| Educational level - primary | -1.46 | 0.23 | 0.19 | 0.28 | -15.57 | <.001 |
| Income - afford when saving | -0.56 | 0.57 | 0.48 | 0.68 | -6.11 | <.001 |
| Income - difficulties paying | -0.88 | 0.41 | 0.34 | 0.51 | -8.17 | <.001 |
| Comorbidities | -0.04 | 0.96 | 0.90 | 1.02 | -1.42 | .156 |
| Living alone | -0.41 | 0.66 | 0.53 | 0.83 | -3.52 | <.001 |
| Village or Small Town (<20,000 Residents) | -0.78 | 0.46 | 0.38 | 0.55 | -8.35 | <.001 |
| Near Visual Impairment x Sex (Women) | 0.11 | 1.12 | 0.84 | 1.48 | 0.76 | .447 |
| Near Visual Impairment x Educational Level (Primary) | -0.03 | 0.97 | 0.73 | 1.29 | -0.19 | .852 |
| Near Visual Impairment x Place of Residence (Village or Small Town (<20,000 Residents) | 0.34 | 1.40 | 1.06 | 1.87 | 2.34 | .019 |
| Near Visual Impairment x Living Arrangements (Alone) | -0.13 | 0.88 | 0.61 | 1.27 | -0.71 | .480 |

**Internet activities: sending, receiving mails**

| Variables | Estimate | OR | lower.CI | upper.CI | Z-value | P-value |
| --- | --- | --- | --- | --- | --- | --- |
| Near visual impairment | -0.32 | 0.73 | 0.53 | 1.00 | -1.96 | .050 |
| Women | -0.14 | 0.87 | 0.70 | 1.08 | -1.25 | .211 |
| Age | -0.11 | 0.90 | 0.88 | 0.91 | -16.85 | <.001 |
| Educational level - primary | -1.94 | 0.14 | 0.11 | 0.19 | -14.35 | <.001 |
| Income - afford when saving | -0.51 | 0.60 | 0.49 | 0.74 | -4.96 | <.001 |
| Income - difficulties paying | -0.85 | 0.43 | 0.33 | 0.55 | -6.41 | <.001 |
| Comorbidities | -0.24 | 0.79 | 0.72 | 0.86 | -5.45 | <.001 |
| Living alone | 0.03 | 1.03 | 0.78 | 1.35 | 0.19 | .848 |
| Village or Small Town (<20,000 Residents) | -0.81 | 0.44 | 0.35 | 0.56 | -6.95 | <.001 |
| Near Visual Impairment x Sex (Women) | 0.07 | 1.07 | 0.74 | 1.55 | 0.38 | .704 |
| Near Visual Impairment x Educational Level (Primary) | -0.11 | 0.90 | 0.57 | 1.41 | -0.46 | .647 |
| Near Visual Impairment x Place of Residence (Village or Small Town (<20,000 Residents) | 0.03 | 1.03 | 0.70 | 1.52 | 0.15 | .877 |
| Near Visual Impairment x Living Arrangements (Alone) | -0.04 | 0.96 | 0.60 | 1.54 | -0.19 | .851 |

**Internet activities: searching for information (e.g. about goods, services)**

| Variables | Estimate | OR | lower.CI | upper.CI | Z-value | P-value |
| --- | --- | --- | --- | --- | --- | --- |
| Near visual impairment | -0.43 | 0.65 | 0.49 | 0.86 | -3.05 | .002 |
| Women | -0.49 | 0.61 | 0.51 | 0.74 | -5.00 | <.001 |
| Age | -0.12 | 0.89 | 0.88 | 0.90 | -22.98 | <.001 |
| Educational level - primary | -1.44 | 0.24 | 0.20 | 0.29 | -14.50 | <.001 |
| Income - afford when saving | -0.35 | 0.70 | 0.59 | 0.85 | -3.72 | <.001 |
| Income - difficulties paying | -0.63 | 0.53 | 0.43 | 0.66 | -5.64 | <.001 |
| Comorbidities | -0.15 | 0.86 | 0.80 | 0.92 | -4.37 | <.001 |
| Living alone | -0.14 | 0.87 | 0.68 | 1.10 | -1.13 | .258 |
| Village or Small Town (<20,000 Residents) | -0.77 | 0.46 | 0.38 | 0.56 | -7.95 | <.001 |
| Near Visual Impairment x Sex (Women) | -0.06 | 0.94 | 0.69 | 1.28 | -0.40 | .692 |
| Near Visual Impairment x Educational Level (Primary) | -0.26 | 0.77 | 0.56 | 1.06 | -1.62 | .105 |
| Near Visual Impairment x Place of Residence (Village or Small Town (<20,000 Residents) | 0.37 | 1.45 | 1.06 | 1.97 | 2.32 | .021 |
| Near Visual Impairment x Living Arrangements (Alone) | 0.20 | 1.22 | 0.82 | 1.81 | 1.00 | .319 |

**Internet activities: Use of online banking**

| Variables | Estimate | OR | lower.CI | upper.CI | Z-value | P-value |
| --- | --- | --- | --- | --- | --- | --- |
| Near visual impairment | -0.21 | 0.81 | 0.58 | 1.13 | -1.22 | .223 |
| Women | -0.36 | 0.70 | 0.55 | 0.88 | -3.06 | .002 |
| Age | -0.13 | 0.88 | 0.87 | 0.89 | -17.59 | <.001 |
| Educational level - primary | -1.93 | 0.15 | 0.11 | 0.19 | -13.53 | <.001 |
| Income - afford when saving | -0.68 | 0.51 | 0.41 | 0.63 | -6.27 | <.001 |
| Income - difficulties paying | -0.99 | 0.37 | 0.28 | 0.49 | -7.08 | <.001 |
| Comorbidities | -0.21 | 0.81 | 0.74 | 0.89 | -4.52 | <.001 |
| Living alone | 0.30 | 1.35 | 1.02 | 1.79 | 2.10 | .036 |
| Village or Small Town (<20,000 Residents) | -0.83 | 0.44 | 0.34 | 0.55 | -6.78 | <.001 |
| Near Visual Impairment x Sex (Women) | 0.10 | 1.11 | 0.74 | 1.64 | 0.47 | .637 |
| Near Visual Impairment x Educational Level (Primary) | -0.59 | 0.55 | 0.32 | 0.95 | -2.16 | .030 |
| Near Visual Impairment x Place of Residence (Village or Small Town (<20,000 Residents) | -0.09 | 0.91 | 0.60 | 1.39 | -0.43 | .666 |
| Near Visual Impairment x Living Arrangements (Alone) | -0.13 | 0.88 | 0.54 | 1.44 | -0.53 | .594 |

**Internet activities: use of instant messaging e.g. Skype, Facetime, Microsoft Messenger**

| Variables | Estimate | OR | lower.CI | upper.CI | Z-value | P-value |
| --- | --- | --- | --- | --- | --- | --- |
| Near visual impairment | 0.04 | 1.04 | 0.74 | 1.47 | 0.24 | .813 |
| Women | -0.02 | 0.98 | 0.77 | 1.25 | -0.16 | .873 |
| Age | -0.09 | 0.91 | 0.90 | 0.93 | -12.65 | <.001 |
| Educational level - primary | -1.01 | 0.36 | 0.28 | 0.48 | -7.40 | <.001 |
| Income - afford when saving | -0.49 | 0.61 | 0.49 | 0.76 | -4.41 | <.001 |
| Income - difficulties paying | -0.55 | 0.58 | 0.44 | 0.76 | -3.98 | <.001 |
| Comorbidities | -0.14 | 0.87 | 0.79 | 0.95 | -3.11 | .002 |
| Living alone | 0.25 | 1.28 | 0.96 | 1.71 | 1.69 | .092 |
| Village or Small Town (<20,000 Residents) | -0.54 | 0.58 | 0.45 | 0.75 | -4.20 | <.001 |
| Near Visual Impairment x Sex (Women) | 0.06 | 1.06 | 0.71 | 1.58 | 0.29 | .773 |
| Near Visual Impairment x Educational Level (Primary) | -0.31 | 0.73 | 0.47 | 1.14 | -1.39 | .164 |
| Near Visual Impairment x Place of Residence (Village or Small Town (<20,000 Residents) | -0.14 | 0.87 | 0.57 | 1.32 | -0.64 | .524 |
| Near Visual Impairment x Living Arrangements (Alone) | -0.04 | 0.96 | 0.60 | 1.55 | -0.15 | .881 |

**Internet activities: participating in chat rooms and using social networking sites e.g. Facebook**

| Variables | Estimate | OR | lower.CI | upper.CI | Z-value | P-value |
| --- | --- | --- | --- | --- | --- | --- |
| Near visual impairment | -0.04 | 0.96 | 0.62 | 1.48 | -0.18 | .855 |
| Women | 0.16 | 1.17 | 0.88 | 1.56 | 1.09 | .275 |
| Age | -0.10 | 0.90 | 0.89 | 0.92 | -10.90 | <.001 |
| Educational level - primary | -0.78 | 0.46 | 0.34 | 0.63 | -4.91 | <.001 |
| Income - afford when saving | -0.27 | 0.76 | 0.58 | 1.00 | -1.94 | .052 |
| Income - difficulties paying | -0.26 | 0.77 | 0.56 | 1.07 | -1.58 | .114 |
| Comorbidities | -0.10 | 0.90 | 0.81 | 1.01 | -1.81 | .071 |
| Living alone | 0.29 | 1.34 | 0.96 | 1.87 | 1.69 | .092 |
| Village or Small Town (<20,000 Residents) | -0.55 | 0.58 | 0.43 | 0.78 | -3.60 | <.001 |
| Near Visual Impairment x Sex (Women) | -0.17 | 0.84 | 0.52 | 1.37 | -0.70 | .483 |
| Near Visual Impairment x Educational Level (Primary) | -0.53 | 0.59 | 0.34 | 1.02 | -1.90 | .058 |
| Near Visual Impairment x Place of Residence (Village or Small Town (<20,000 Residents) | 0.13 | 1.14 | 0.69 | 1.89 | 0.50 | .617 |
| Near Visual Impairment x Living Arrangements (Alone) | 0.07 | 1.07 | 0.60 | 1.91 | 0.23 | .820 |

**Internet activities: searching for healthcare information**

| Variables | Estimate | OR | lower.CI | upper.CI | Z-value | P-value |
| --- | --- | --- | --- | --- | --- | --- |
| Near visual impairment | -0.21 | 0.81 | 0.60 | 1.09 | -1.38 | .168 |
| Women | 0.01 | 1.01 | 0.83 | 1.24 | 0.07 | .944 |
| Age | -0.10 | 0.90 | 0.89 | 0.92 | -16.68 | <.001 |
| Educational level - primary | -1.25 | 0.29 | 0.23 | 0.36 | -11.26 | <.001 |
| Income - afford when saving | -0.06 | 0.94 | 0.77 | 1.15 | -0.60 | .548 |
| Income - difficulties paying | -0.34 | 0.71 | 0.56 | 0.91 | -2.78 | .005 |
| Comorbidities | -0.02 | 0.98 | 0.91 | 1.05 | -0.63 | .525 |
| Living alone | -0.03 | 0.97 | 0.75 | 1.25 | -0.24 | .811 |
| Village or Small Town (<20,000 Residents) | -0.58 | 0.56 | 0.46 | 0.69 | -5.58 | <.001 |
| Near Visual Impairment x Sex (Women) | 0.00 | 1.00 | 0.71 | 1.40 | 0.02 | .985 |
| Near Visual Impairment x Educational Level (Primary) | -0.47 | 0.63 | 0.43 | 0.91 | -2.47 | .014 |
| Near Visual Impairment x Place of Residence (Village or Small Town (<20,000 Residents) | 0.08 | 1.08 | 0.77 | 1.53 | 0.44 | .663 |
| Near Visual Impairment x Living Arrangements (Alone) | 0.07 | 1.07 | 0.70 | 1.64 | 0.33 | .740 |

**Internet activities: shopping online, ordering tickets, e.g. cinema, theater, train, airline tickets**

| Variables | Estimate | OR | lower.CI | upper.CI | Z-value | P-value |
| --- | --- | --- | --- | --- | --- | --- |
| Near visual impairment | -0.04 | 0.96 | 0.65 | 1.43 | -0.18 | .859 |
| Women | -0.23 | 0.79 | 0.61 | 1.04 | -1.68 | .093 |
| Age | -0.10 | 0.90 | 0.89 | 0.92 | -11.53 | <.001 |
| Educational level - primary | -1.62 | 0.20 | 0.14 | 0.28 | -9.21 | <.001 |
| Income - afford when saving | -0.45 | 0.64 | 0.50 | 0.82 | -3.62 | <.001 |
| Income - difficulties paying | -0.92 | 0.40 | 0.28 | 0.56 | -5.27 | <.001 |
| Comorbidities | -0.18 | 0.84 | 0.75 | 0.93 | -3.17 | .002 |
| Living alone | 0.00 | 1.00 | 0.71 | 1.41 | 0.01 | .991 |
| Village or Small Town (<20,000 Residents) | -0.13 | 0.88 | 0.67 | 1.16 | -0.96 | .336 |
| Near Visual Impairment x Sex (Women) | -0.12 | 0.89 | 0.55 | 1.43 | -0.50 | .618 |
| Near Visual Impairment x Educational Level (Primary) | 0.07 | 1.07 | 0.60 | 1.93 | 0.24 | .811 |
| Near Visual Impairment x Place of Residence (Village or Small Town (<20,000 Residents) | -0.51 | 0.60 | 0.36 | 0.99 | -2.01 | .045 |
| Near Visual Impairment x Living Arrangements (Alone) | -0.51 | 0.60 | 0.30 | 1.20 | -1.46 | .144 |

**Internet activities: government websites for electronic handling of government inquiries (e-administration)**

| Variables | Estimate | OR | lower.CI | upper.CI | Z-value | P-value |
| --- | --- | --- | --- | --- | --- | --- |
| Near visual impairment | -0.18 | 0.84 | 0.52 | 1.34 | -0.73 | .465 |
| Women | -0.40 | 0.67 | 0.48 | 0.94 | -2.28 | .023 |
| Age | -0.11 | 0.90 | 0.88 | 0.92 | -10.05 | <.001 |
| Educational level - primary | -2.01 | 0.13 | 0.08 | 0.23 | -7.59 | <.001 |
| Income - afford when saving | -0.50 | 0.61 | 0.45 | 0.81 | -3.34 | .001 |
| Income - difficulties paying | -1.14 | 0.32 | 0.20 | 0.50 | -4.99 | <.001 |
| Comorbidities | -0.10 | 0.90 | 0.79 | 1.03 | -1.47 | .143 |
| Living alone | 0.11 | 1.12 | 0.73 | 1.71 | 0.52 | .603 |
| Village or Small Town (<20,000 Residents) | -0.43 | 0.65 | 0.46 | 0.93 | -2.35 | .019 |
| Near Visual Impairment x Sex (Women) | 0.06 | 1.06 | 0.59 | 1.90 | 0.20 | .844 |
| Near Visual Impairment x Educational Level (Primary) | 0.02 | 1.02 | 0.43 | 2.40 | 0.04 | .970 |
| Near Visual Impairment x Place of Residence (Village or Small Town (<20,000 Residents) | -0.07 | 0.93 | 0.50 | 1.73 | -0.24 | .812 |
| Near Visual Impairment x Living Arrangements (Alone) | -0.08 | 0.92 | 0.43 | 2.00 | -0.21 | .830 |
